# Resveratrol analog, triacetylresveratrol, a potential immunomodulator of lung adenocarcinoma immunotherapy combination therapies

**DOI:** 10.3389/fonc.2022.1007653

**Published:** 2023-02-09

**Authors:** Jian He, Nianxiang Qiu, Xianchao Zhou, Mei Meng, Zixue Liu, Jingquan Li, Shiyu Du, Zhiqiang Sun, Hui Wang

**Affiliations:** ^1^ State Key Laboratory of Oncogenes and Related Genes, Center for Single-Cell Omics, School of Public Health, Shanghai Jiao Tong University School of Medicine, Shanghai, China; ^2^ Department of Interventional Radiology, The Tumor Hospital of Jilin Province, Changchun, China; ^3^ Engineering Laboratory of Nuclear Energy Materials, Ningbo Institute of Materials Technology and Engineering, Chinese Academy of Sciences, Ningbo, Zhejiang, China; ^4^ Key Laboratory of Superlight Materials and Surface Technology, Ministry of Education, College of Materials Science and Chemical Engineering, Harbin Engineering University, Harbin, China

**Keywords:** SIRT2, lung adenocarcinoma, biomarker, tumor infiltration lymphocytes, prognosis

## Abstract

**Introduction:**

Resveratrol, an activator for longevity regulatory genes-sirtuin family (SIRTs) and Sirtuin 2 (SIRT2) is an important factor of SIRTs which demonstrated biological function in cancers, but the underlying mechanism is unrevealed.

**Methods:**

We investigated the mRNA and protein levels of SIRT2 in a variety of cancers and the potential role for clinical prognosis, as well as analysed the association between the gene and immune infiltration in various cancers. And an analysis of two types of lung cancer was conducted to construct a systematic prognostic landscape. Finally, putative binding site of the triacetylresveratrol bound to SIRT2 was built from homology modeling.

**Results and discussion:**

We concluded that higher mRNA and protein levels of SIRT2 affected prognosis in various types of cancers, especially in LUAD cohorts. In addition, SIRT2 is linked with a better overall survival (OS) in LUAD patients. Further research suggested a possible explanation for this phenotype might be that SIRT2 mRNA levels are positively correlated with infiltrating status of multiple immunocytes in LU-AD but not LUSC, i.e. SIRT2 expression may contribute to the recruitment of CD8+T cell, CD4+ T cell, T cell CD4+ memory resting, Tregs, T cell NK and positively correlated to the expression of PD-1, also excluding neutrophil, T cell CD8+ naïve and B cell plasma cells in LUAD. We found that triacetyl-resveratrol demonstrated the most potent agonist efficiency to SIRT2 and the EC 50 as low as 142.79 nM. As a result, SIRT2 appears to be a promising novel biomarker for prognosis prediction in patients with LUAD and triacetylresveratrol might be a potential immunomodulator of LUAD to anti-PD-1 based immunotherapy combination therapies.

## Introduction

Lung cancer is one of the most common malignancies worldwide (1). It is the most prevalent malignancy in China and the second most common malignancy in the United States, as well as being the leading cause of cancer deaths in both countries (2). Scientists and doctors have made major breakthroughs in the treatment of various types of lung cancer, but there is still a long way to go. According to histopathology, lung cancer can be divided into two subtypes, small cell lung cancer (SCLC) and non-small cell lung cancer (NSCLC) (3). NSCLC is the most common subtype of lung cancer and can be further diagnosed as subtypes such as lung squamous cell carcinoma (LUSC) and lung adenocarcinoma (LUAD) (4). While earlier stages of NSCLC are treated with surgery, advanced stages of the NSCLC are normally treated with chemotherapy or chemotherapy combined with radiotherapy (4). However, despite these treatment procedures, relapse, metastasis, and drug resistance after treatment continue to result in a poor prognosis for patients, with an overall 5-year survival rate of only 16.6% across all stages (4).

Over the past 20 years, NSCLC was once considered to be a non-immunogenic disease. However, a growing number of research on tumor immune interactions has argued against this model in lung cancer and a number of other types of cancer.

Based on the fact that tumorigenesis and tumor development are closely linked to immune-related interaction mechanisms, immunotherapy shows a broad prospect of clinical application in cancer treatment, and scientists are attempting to harness the body’s own immune system to fight and defeat malignant tumors (4). More recently, immunotherapy, including the use of adoptive cellular therapy, monoclonal antibodies and tumor vaccines, has been used in the clinical treatment of many types of cancer with a promising effectiveness, such as melanoma and lung cancer (5).

In recent years, the discovery of immune checkpoints and the development of their inhibitors have revolutionized the treatment of NSCLC, including programmed cell death protein-1 (PD-1) and programmed cell death protein ligand-1 (PD-L1). Meanwhile, therapies targeting both of these immune checkpoints have shown promising anti-tumor effects in several other cancers (6).

An increasing number of studies have investigated the role of tumor-infiltrating lymphocytes (TILs) in regulating chemotherapy response and clinical prognosis in a variety of cancers, for example, tumor-infiltrating neutrophils (TINs) and tumor-associated macrophages (TAMs) have been reported to be associated with prognosis (7). Therefore, it is urgent and imperative to determine immunophenotypes of tumor immune interactivity and identify new immune therapy targets for lung cancer patients (8, 9).

Longevity regulatory genes, sirtuin family, have been identified in many eukaryotes (10). Sirtuin 2 (SIRT2) is a member of the sirtuin family homologous to the yeast Sir2, which is a Class III histone deacetylase (HDACs) primarily found in the cytosol (11). SIRT2 regulates a variety of physiological processes by participating in the deacetylation of histones and some nonhistone, and is thought to be of undisputed importance in carcinogenesis, but there is much debate as to whether it is an oncogene or a tumor suppressor (12, 13). There is considerable inconsistency among studies on the relationship between NSCLC and SIRT2, but the existing studies are mainly based on cell lines analysis (14, 15), and SIRT2 is not yet known to be associated with clinical outcomes of NSCLC, nor with the mechanism responsible for it.

Resveratrol, a phytoalexin produced by vine, has been shown potent activity in the sirtuin family. Resveratrol increases DNA stability and extends lifespan by 70% by enhancing Sir2 to mimic calorie restriction in yeast (16, 17). Therefore, scientists believed this phenomenon signals new directions for the use of sirtuin activators, especially in cancer therapeutics (18, 19).

## Materials and methods

### Ethics approval

This project was permitted and under the supervision of the Ethics Committee of Shanghai Jiao Tong University School of Medicine.

### SIRT2 gene expression level analysis

Different databases were utilized to investigate the SIRT2 mRNA expression level in different cancer types, including TIMER 2.0, UALCAN, and GEPIA2 (20–22), which are webservers for visualization of TCGA data. In this study, we set the following thresholds: *P*-value of 10^-6^, a fold change of 2, and a gene ranking in the top 5%.

### Prognosis analysis

Oncolnc, GEPIA2, and PrognoScan databases were applied to study the association between SIRT2 expression and survival rates in different types of cancer (23, 24), which searching for relationships between gene expression levels and patient outcomes, including overall survival (OS) and disease-free survival (DFS), by analyzing a large collection of microarray data. Based on the Cox p-value, the threshold was adjusted by 0.05. Moreover, Human Protein Atlas (HPA) Version 21.0 was used to determine the correlation between SIRT2 protein levels and survival rate as well as different cancer staging in LUAD and LUSC (25). The p-values of the log-rank test and the HRs with 95% confidence intervals (Cls) were analyzed.

### Clinical parameters analysis

The association of SIRT2 expression and clinicopathological parameters, including cancer stages, patient race, gender, age, and smoking habit were analyzed by UALCAN and MEXPRESS (26) platform, which is used for integrating and visualizing clinical, expression and methylation data in TCGA at the single-gene level.

### Methylation analysis

The MEXPRESS platform was accessed to identify the methylation level in promoter region SIRT2. Utilizing UALCAN, we assessed the level of methylation and expression, as well as the survival of a specific target gene across several clinicopathological features, including stages and age. T-tests were performed to compare statistical significance.

### Biological network analysis

GeneMANIA is identified single genes related to a set of input genes (27) to construct the SIRT2 biological network based on a set of function-association data, including co-expression, genetic, and protein interaction pathways, colocalization, and protein domain homology.

### LinkedOmics analysis

The LinkedOmics database contains data from the TCGA covering 32 types of cancer and more than 10,000 patients (28). LinkFinder was used to identify the differentially expressed genes (DEGs) in TCGA. Pathways and networks were identified using LinkInterpreter.

### Immune infiltrates level and gene correlation analysis

Various types of malignancy were examined for SIRT2 expression, as well as its association with immune-infiltrating cells, such as T cells (CD4+ T cells, CD8+ T cells), B cells, macrophages, neutrophils, and DCs. Additionally, correlation modules were used to examine the associations between SIRT2 expression levels and markers of TIICs. The TIIC marker genes include markers for T cells (CD8+ T cells, general T cells), B cells, TAMs, monocytes, macrophages (type 1 macrophages, M1 and Type 2 macrophages, M2), neutrophils, natural killer (NK) cells, dendritic cells (DCs), T-helper cells (Th1, Th2 and Th17), follicular helper T cells (Tfh), T regulatory cells (Tregs) and exhausted T cells. And our previous publication referenced the gene marker sets (29–31). Gene expression levels were determined by calculating log2 RSEM.

### Molecular docking study

To clarify the SIRT2 binding mode further, we performed a docking simulation. The molecular docking was carried out with AutoDock 4.2.6 software (32) to determine which compound best matched the crystal structure of the enzyme. In order to accomplish docking, first, the crystal structure of SIRT2 (PDB code: 5DY5) (33) was obtained from Protein Data Bank (PDB) (34) and then PyMOL 2.5 (35) was used to process the structures and delete unnecessary ligands. In order to validate the docking results, we extracted the co-crystallized ligand from the protein and re-docked it into the same position. Docking simulations were performed with the ligands fully flexible while the receptor residues were assumed to be rigid. Both the crystal enzyme structures and compounds were constructed with AutoDock Tools 1.5.7. As targets for the enzymes, hydrogen atoms with polarity, Kollman united atoms with type charge, and Gasteiger partial charges were added. The compounds were prepared by adding Gasteiger partial charges, combining non-polar hydrogen atoms, and defining rotatable bonds as a less conformational explosion. AutoGrid was used to generate grid maps and spacing.

### Statistical analysis

A log-rank test is used to determine the impact of Kaplan–Meier plots, GEPIA, and PrognoScan on HR and *p* or Cox *p* values. In addition, Gene expression correlation coefficients were evaluated using Spearman rank correlations, with P < 0.05 considered statistically significant.

## Results

### The expression levels of SIRT2 in different human cancers

**Figure 1:**
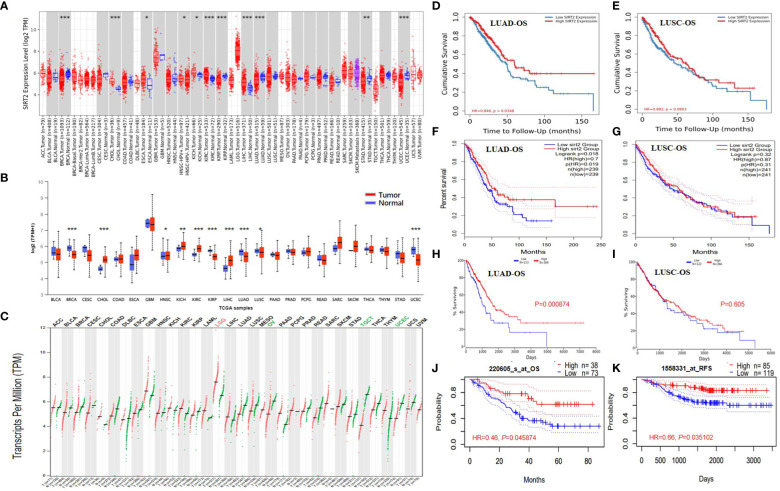
SIRT2 expression levels in different types of human cancers in different databases. Survival curves comparing the high and low expression of SIRT2 in LUAD and LUSC. SIRT2 expression profile across all tumor samples and paired normal tissues (Dot plot) *via* TIMER2.0 **(A)** and UALCAN **(B)**. *P-*value Significant Codes: 0 ≤ *** < 0.001 ≤ ** < 0.01 ≤ * < 0.05 ≤. < 0.1. The threshold was set as follows: *P*-value of 1E-6, fold change of 2, and gene ranking top 5%. SIRT2 expression profile across all tumor samples and paired normal tissues *via* GEPIA 2.0 **(C)**, each dots represent the expression of samples. The gene expression profile across all tumor samples and paired normal tissues (Bar plot). The height of bars represents the median expression of certain tumor type or normal tissue (each dot representing a distinct tumor or normal sample). Overall survival curves comparing the high and low expression of SIRT2 in LUAD **(D)** and LUSC **(E)** in the TIMER2.0. Overall survival curves comparing the high and low expression of SIRT2 in LUAD **(F)** and LUSC **(G)** in the GEPIA. A comparison of overall survival curves in Oncolnc database for LUAD **(H)** and LUSC **(I)** based on SIRT2 expression levels. The PrognoScan database compares high and low expressions of SIRT2 in LUAD based on OS **(J)** and Distant Metastatic Free Survival **(K)** curves.

The present study investigated the expression levels of SIRT2 in human normal and cancer tissues, using the dominant online database TIMER2.0 and GEPIA2. Compared with adjacent normal tissues (cancer vs. normal), we found that SIRT2 was highly expressed in CHOL, ESCA, KICH, KICP, LIHC and conversely low expressed in BRCA, KIRP, LUAD, LUSC, STAD, UCEC (**Figure 1A**). In **Figures 1B, C**, we show the differences in SIRT2 mRNA levels across all TCGA tumor tissue in comparison to matched normal tissue and GTEx data. Compared with adjacent normal tissues, a significant decrease in SIRT2 expression was observed in tumor tissue of BRCA, KIRP, LUAD, LUSC, and UCEC, which are consistent with the TIMER2.0 database, while SIRT2 expression was significantly lower in OV, TGCT and UCEC tumor tissues and higher in LGG compared to adjacent normal tissues *via* GEPIA2 (**Figure 1C**).

### Prognostic potential of SIRT2 in various types of cancer

We analyzed the relationship between SIRT2 expression levels and prognosis in different cancer populations. The effect of SIRT2 expression levels on patient survival was assessed using the PrognoScan tool. **Supplementary Table S1** describes the detailed relationship between the expression levels of SIRT2 and the prognostic potential of various cancers. In particular, the expression level of SIRT2 affected OS in brain cancer (OS HR = 0.31, 95% CI = 0.15 to 0.64, Cox *P* = 0.001496), lung cancer significantly (OS HR = 0.58, 95% CI = 0.38 to 0.87, Cox *P* = 0. 0.009042), moreover SIRT2 also affected relapse free survival of lung cancer (OS HR = 0.66, 95% CI = 0.46 to 0.94, Cox *P* = 0.020039) (**Supplementary Table S1**). It is therefore plausible that high SIRT2 expression is an independent risk factor and is associated with a better prognosis for lung cancer patients, while a hazard ratio (HR) of less than 1 suggests a protective effect of SIRT2.

Of particular interest to us is the fact that adenocarcinomas and squamous cell carcinomas of the lung show different patterns of correlation (**Figures 1D-K**). The lung adenocarcinoma cohort based on TCGA *via* TIMER2.0, GEPIA, and UALCAN (**Figures 1D, F, H**) and (GSE31210) (**Figure 1J**) demonstrated that higher SIRT2 expression level correlates with better OS, and better relapse-free survival (RFS) (**Figure 1K**) but there is nonsignificant different in all lung squamous cell carcinoma cohorts *via* various databases (**Figures 1E, G, I**). Based on these data, we revealed the prognostic value of SIRT2 for several types of lung cancer, i.e., higher or lower SIRT2 expression varies in prognostic value with each type.

### Different clinical characteristics of lung cancer are influenced by SIRT2 expression levels

**Figure 2:**
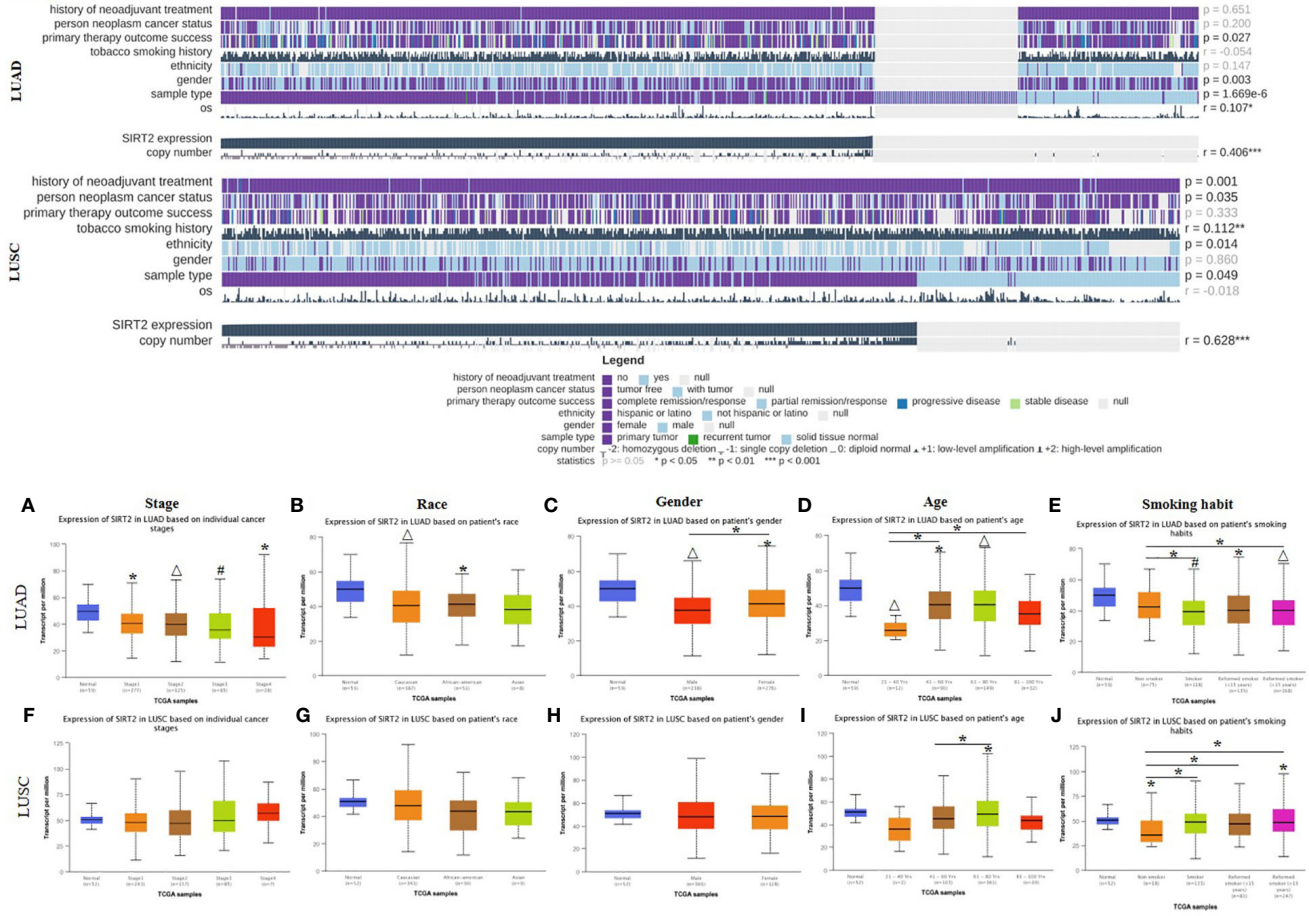
TCGA data visualization of the SIRT2 affects on clinicopathological parameters in LUAD and LUSC cohorts. SIRT2 expression levels influence the clinicopathological parameters in LUAD and LUSC cohorts using MEXPRESS (upper), data were reordered by the expression of SIRT2. Expression levels of SIRT2 *i*mpacts the individual cancer stages **(A, F)**, patient’s race **(B, G)**, patient’s gender **(C, H)**, patient’s age **(D, I)**, patient’s smoking habit **(E, J)** in LUAD and LUSC cohorts using UALCAN (lower). *P ≤ 0.05, #*P ≤* 0.001, △*P ≤* 0.0001.

By investigating the association between SIRT2 expression levels and different clinical features, we expect to reveal the mechanism and relevance of SIRT2 expression levels in various cancer types, especially in patients with different clinical stages of lung cancer. We found that the SIRT2 expression level was related to different clinical covariates, including the history of neoadjuvant therapy, person neoplasm cancer status, primary therapy outcome success, ethnicity, and some other clinical characteristics in LUAD and LUSC cohorts using MEXPRESS database (**Figure 2** upper). We also found that high levels of SIRT2 expression in the early stages of LUAD may predict better OS, whereas no such association was found in the LUSC patient cohort (**Table 1**). Also, the expression level of SIRT2 varies significantly between races and genders of LUAD rather than in LUSC (**Figures 2A-C, F-H**). Moreover, the expression level of SIRT2 demonstrated an age-depended pattern in LUAD rather than in LUSC (**Figures 2D, I** and **Supplementary Table 2**). Together, these arrestive phenomena and the differential survival correlation between LUAD and LUSC in **Figure 1** may indicate a potential association between SIRT2 expression and the prognosis of different cancer types.

**Table 1:** Correlation of the mRNA expression level of SIRT2 in different stage and clinical prognostic potential in lung cancer with different clinicopathological factors.

Clinicopathological Characteristics	Overall Survival						
	LUAD (*n* = 513)				LUSC (*n* = 501)		
	N		Hazard Ratio	*p*-Value	N	Hazard Ratio	*p*-Value
**Gender**							
Female	274		0.64 (0.43 – 0.96)	**0.031**	129	0.73 (0.41 – 1.31)	0.29
Male	234		0.48 (0.31 – 0.74)	**0.00073**	366	0.67 (0.47 – 0.95)	**0.024**
**Race**							
White	387		0.52 (0.37 – 0.74)	**0.00018**	348	0.8 (0.57 – 1.13)	**0.2**
Asian	—		—	**—**	—	—	**—**
Black/African American		52	0.44 (0.14 – 1.38)	0.15	29	0.44 (0.17 – 1.11)	0.075
**Mutation burden**							
High	255		0.42 (0.26 – 0.7)	**0.00051**	240	0.62 (0.42 – 0.92)	**0.017**
Low	244		0.71 (0.47 – 1.07)	0.099	242	0.75 (0.49 – 1.17)	0.2
**Stage**							
1	270		0.59 (0.35 - 1)	**0.049**	242	0.65 (0.41 – 1.05)	0.074
2	119		0.5 (0.29 – 0.88)	**0.014**	159	0.53 (0.32 – 0.88)	**0.014**
3	81		0.41 (0.23 – 0.74)	**0.0024**	83	0.64 (0.31 – 1.32)	0.23
4	26		1.8 (0.61 – 5.31)	0.28	0	—	—

Bold values indicate p < 0.05.

### Low promoter methylation levels of SIRT2 impact the clinicopathological parameters of lung cancer patients

**Figure 3:**
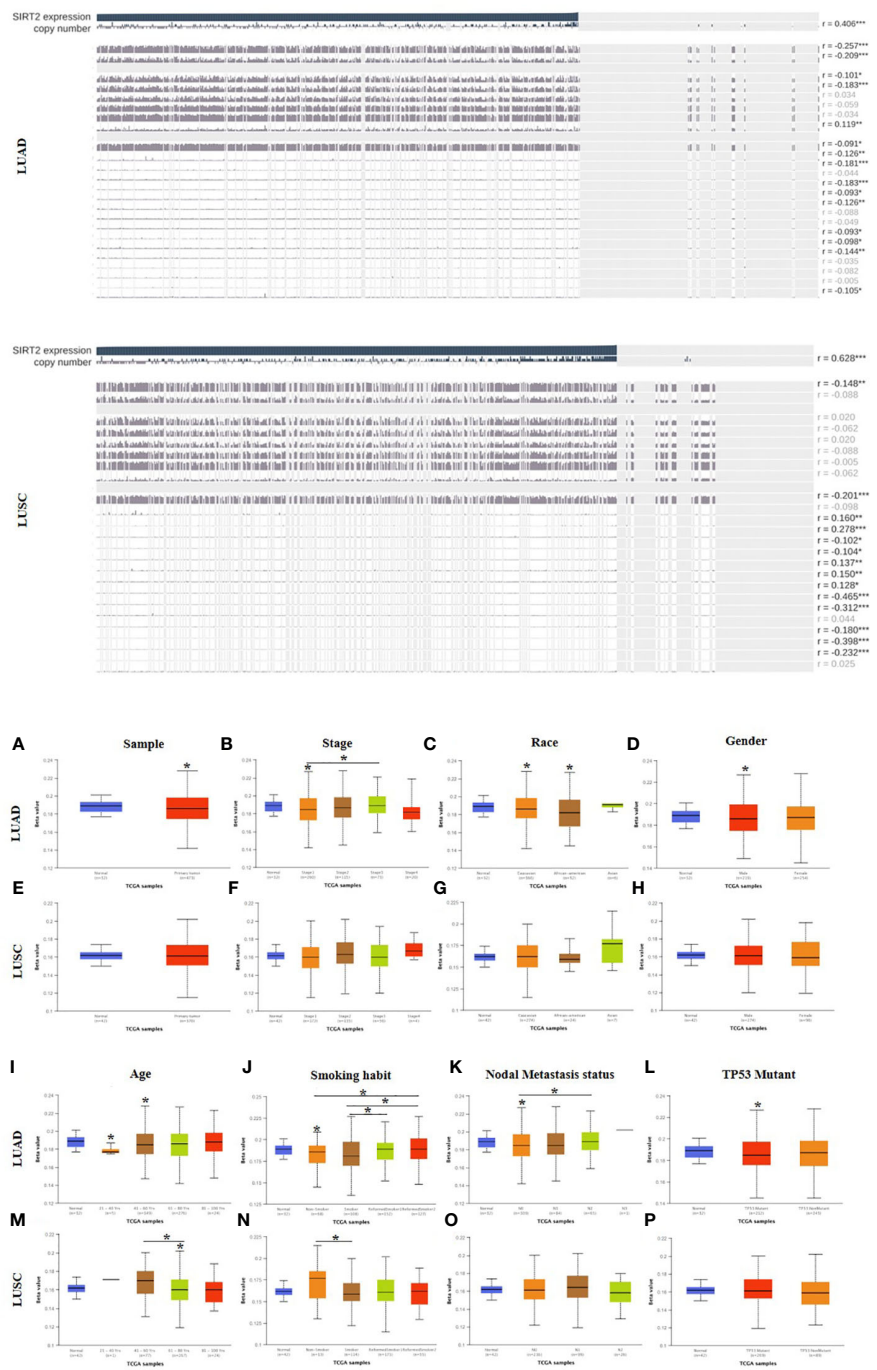
Visualization of SIRT2 promoter methylation levels influence the clinicopathological parameters among LUAD and LUSC patient cohorts. Methylation sites prediction using MEXPRESS (upper). Promoter methylation levels of SIRT2 in sample types **(A, E)**, stage **(B, F)**, race **(C, G)**, gender **(D, H)**, age **(I, M)**, smoking habit **(J, N)**, nodal metastasis status **(K, O)** and TP53 mutant **(L, P)** in LUAD and LUSC cohorts respectively using UALCAN (lower). *p ≤ 0.05.

To unravel the mechanism underlying the correlation between SIRT2 expression and prognostic factors in LUAD and LUSC, we analyzed the methylation sites and methylation status of SIRT2 in tumor and normal tissues of LUAD and LUSC cohorts respectively *via* MEXPRESS (**SI-Table 3** and **Figure 3**). The different 5 CpG methylation sites between LUAD and LUSC were listed in **SI-Table 3**. Among these CPG locations, CPG 38899816 attracted our attention, since SIRT2 level is significantly positively correlated with the methylation status in LUAD cohort, whereas in the LUSC cohort there was an opposite pattern of correlation. We found that 5 CpG sites had significantly lower hypermethylation in the LUAD and LUSC tumor samples than in the normal tissues (P< 0.0001, **Figures 3A, E**). An increasing promoter methylation tendency of SIRT2 was detected from early to late stages in LUAD, which could be an explanation for the lower level of SIRT2 expression was associated with the earlier stages of the progress of LUAD rather than that of LUSC. (**Figures 3A, E**). It was interesting to find that the same pattern was found in the analysis of nodal metastasis, which suggests that SIRT2 promoter methylation is correlated with the nodal metastatic formation in the later phase (**Figures 3K, O**). The findings of promoter methylation here may provide insight into how SIRT2 expression levels fluctuate during lung cancer progression.

### Crucial roles for SIRT2 in immune system activation

**Figure 4:**
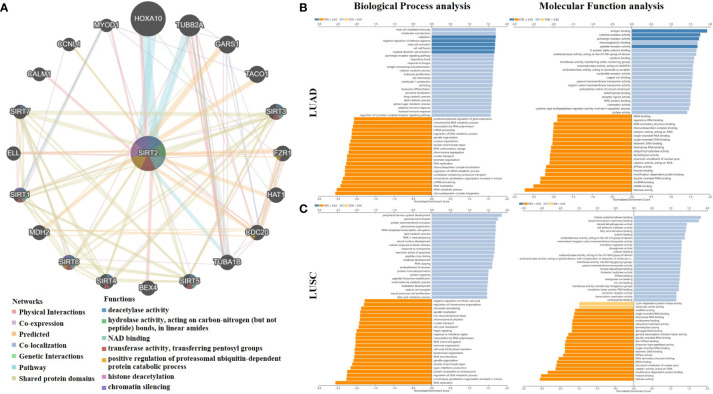
SIRT2-related gene enrichment analysis by using GENEMANIA and LinkedOmics. **(A)** A TCGA SIRT2 interaction network. Lines in different colors represent different bioinformatics methods, and rings in different colors represent different gene functions. Analysis of SIRT2 correlated genes in LUAD **(B)**, LUSC **(C)** using the Gene Ontology for Biological Process and Molecular Function. In dark blue and orange, FDR is less than 0.05, in light blue and orange, FDR is greater than 0.05. FDR stands for false discovery rate.

**Figure 4A** shows 20 SIRT2-related proteins based on the analysis of physical interaction, co-expression, predicted, co-localization, genetic interactions, pathway, and shared protein domain that were screened through the GeneMANIA database. We found that SIRT2 has a high correlation with the SIRT family and FZR1, CDC20 in protein deacetylase activity, hydrolase activity, NAD binding, transferase activity, histone deacetylation, and chromatin silencing, etc. functions (**Figure 4A**). A gene set enrichment analysis revealed that SIRT2-associated DEGs are involved in a variety of immunobiological processes in LUAD, such as ‘sialylation’, ‘mast cell activation’, ‘myeloid dendritic cell activation, whereas totally differently in LUSC (**Figures 4B, C**). Moreover, we found that the immuno-associated molecular functions such as ‘antigen binding’, ‘cytokine receptor activity’, ‘immunoglobulin binding’, and ‘purinergic receptor activity’ were involved in LUAD rather than in LUSC cohorts (**Figures 4B, C**). In light of all these findings, SIRT2 seems to be important for immune system activation, cellular responses to stimulation, and a number of other functions.

### SIRT2 expression level is correlated with LUAD immune infiltration status

**Figure 5:**
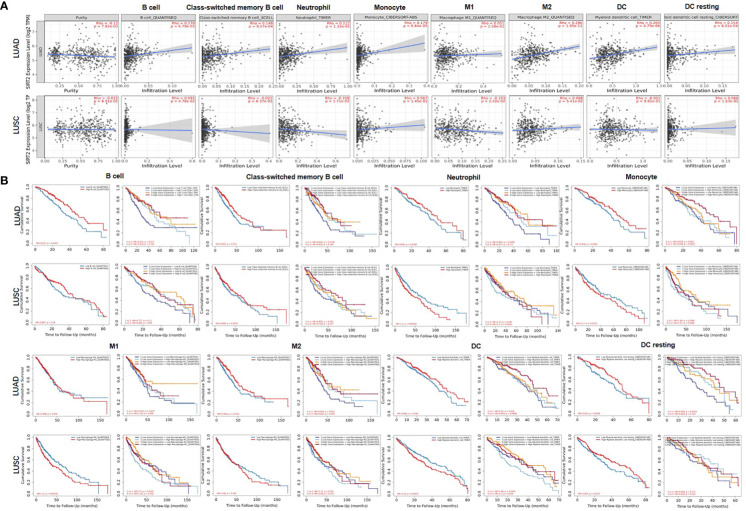
Correlation between SIRT2 expression and immune infiltration level in **(A)** LUAD and **(B)** LUSC. **(A)** SIRT2 expression was strongly negatively correlated with tumor purity but strongly positively correlated with the level of B cells, Class-switched memory B cell, neutrophils, monocyte, M1, M2 and DCs in LUAD (*n* = 515). **(B)** SIRT2 expression is weak positive and significantly negative correlations with infiltrating levels of B cell, neutrophils and M1 in LUSC but a significant correlation was not found between SIRT2 expression and the infiltrating levels of B cells, CD8+ T cells, macrophages and DCs (*n* = 501).

We examined the correlation between the expression levels of SIRT2 and the level of immune infiltration in both types of lung cancer. Significantly positive correlations have been observed between the SIRT2 expression level and infiltrating levels of B cell, class-switched memory B cell, neutrophil, monocyte, M2, DC, and DC resting cells in LUAD rather than in LUSC (**Figure 5** upper).

It is interesting to note that SIRT2 expression is positively correlated with better OS and higher immune-infiltration rates in LUAD rather than in LUSC. Based on these findings, SIRT2 may be involved in immune infiltration in different types of lung cancer and promote a better prognosis in LUAD instead of in LUSC. Another phenomime that attracted our attention is that the higher proportion of these types of cell above, the better OS in LUAD (**Figure 5** lower). Moreover, the expression level of SIRT2 is correlated to these cell types and correlated with significantly higher OS in LUAD rather than in LUSC (**Figure 5** lower). Furthermore, we studied the subtypes of the T cell, including CD8+T, CD4+ T cells, T cell CD4+ memory resting, Tregs, and T cell NK, and we found similar patterns of the correlations in LUAD (**Supplementary Figure 1**).

### Different correlation patterns between tumor and normal tissue in LUAD patients

More interestingly, the infiltration levels of most of these immune cells above were strongly correlated to the expression of SIRT2 in tumor tissues of LUAD patients.

**Figure 6:**
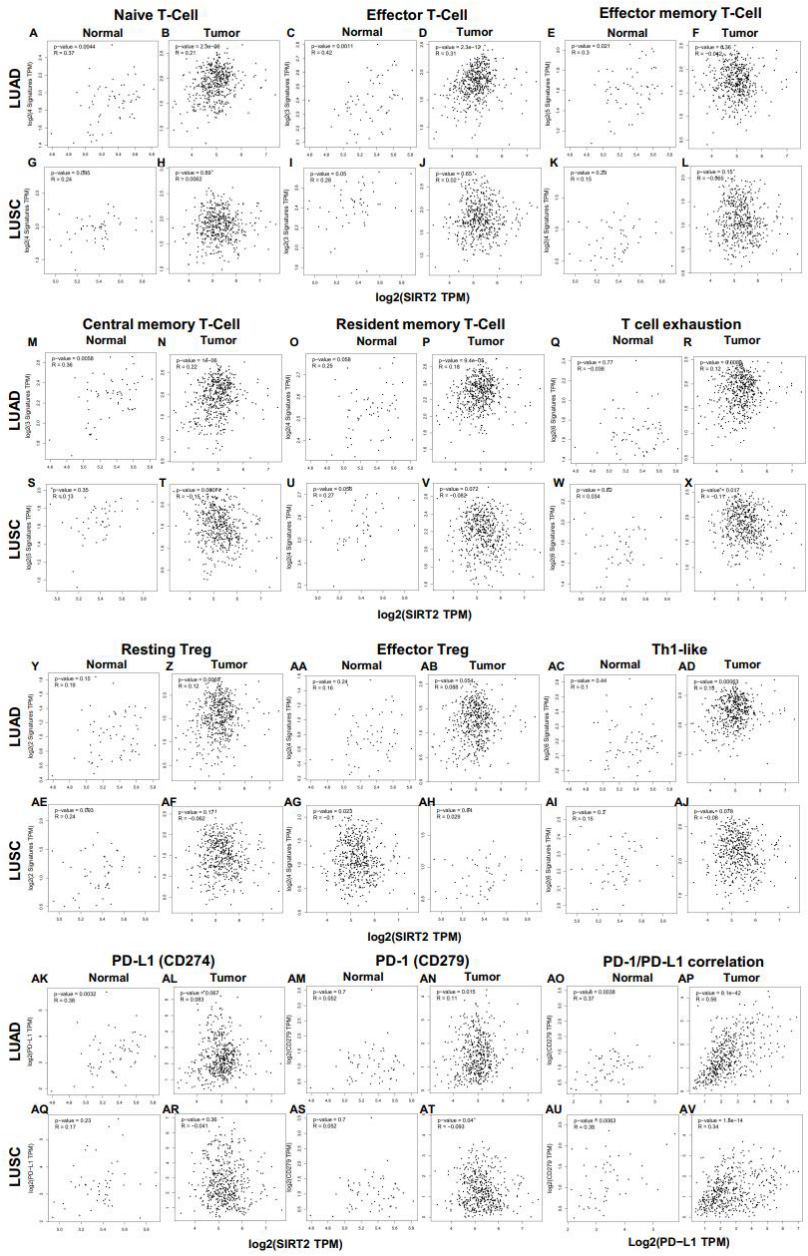
Correlation between SIRT2 expression and various immune cells and PD-1/PD-L1 in normal and tumor tissue of LUAD and LUSC. **(A-R)** Scatterplots of correlations between SIRT2 expression and naive T-Cell **(A, B)**, effector T-Cell **(C, D)**, effector memory T-Cell **(E, F)**, central memory T-Cell **(M, N)**, resident memory T-Cell **(O, P)**, T cell exhaustion **(Q, R)**, resting Treg **(Y, Z)**, effector Treg **(AA, AB)**, Th1-like **(AC, AD)** cells and PD-1/PD-L1 axis **(AK-AP)** in the normal and tissue of LUAD; Scatterplots of correlations between SIRT2 expression and gene markers of naive T-Cell **(G, H)**, effector T-Cell **(I, J)**, effector memory T-Cell **(K, L)**, central memory T-Cell **(S, T)**, resident memory T-Cell **(U, V)**, T cell exhaustion **(W, X)**, resting Treg **(AE, AF)**, effector Treg **(AG, AH)**, Th1-like **(AI, AJ)** cells and PD-1/PD-L1 axis **(AQ-AV)** in LUSC.

However, no significant correlation was found between SIRT2 and immune cells in the LUSC cohort (**Figure 6** and **Supplementary Figure 2**). Our findings suggest a distinct pattern of correlation between tumor and normal tissue in LUAD patients. This exciting and innovative research suggests that SIRT2 may regulate various types of T cell (naive T cell, effector T cell, central memory T cell) as well as Treg activation and recruitment in LUAD, and SIRT2 may be a new clinical therapeutic target for LUAD (**Figures 6A-D** and **Supplementary Figures 2E, F**). LUAD patients with a high level of SIRT2 expression have a high level of effector memory T cell, resident memory T cell, T cell exhaustion, resting Treg, Th1, monocytes, and DCs infiltrating into the tumor tissue (**Figures 6Z, R, AD**, and **Supplementary Figures 2O, Q**). In addition, these results further shed light on the close relationship between SIRT2 and TILs. ()

### Resveratrol analog, triacetylresveratrol, demonstrated potent binding efficiency to SIRT2

**Figure 7:**
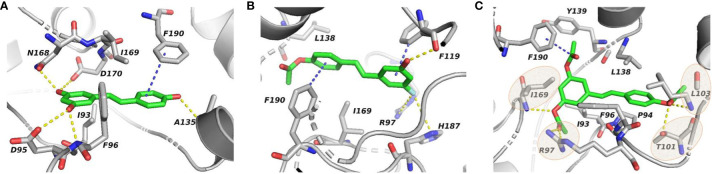
Ligand-binding pocket in SIRT2 for Resveratrol **(A)**, Resveratrol analog 2 **(B)**, and Resveratrol analog 10 **(C)**, respectively. Gray cartoon representations are used for the receptor. Sticks represent resveratrol and its analogs (green carbons) as well as receptor residues (grey carbons) involved in ligand binding.

In order to figure out more efficient compounds than resveratrol, we studied the binding efficiency of dihydroresveratrol and seven other resveratrol analogs (analog 1, 2, 10, 28, 31, 36, 39) (**Table 1**). And we found the analog 10, that is triacetylresveratrol (CAS no. 42206-94-0), which showed the lowest binding energy, that is, the highest binding affinity (**Table 1**). The EC50 of triacetylresveratrol is 241.84 nM, which is almost an eighth of Resveratrol (1.92 uM) and Resveratrol analog 1 (1.73 uM). Among these chemicals, Dihydroresveratrol with the highest binding energy, which suggests it is the least active compound. To elucidate the mechanism involved, we demonstrated the ligand-binding pocket in SIRT2 for Resveratrol and the analogs (**Figure 7**), and in this way, we found a series of differences in binding affinity and sites among Resveratrol, analog 2 and 10.

## Discussion

SIRT2 is a member of the sirtuin family and there are seven of them, SIRT1 to SIRT7 in mammals (36). SIRT1 is the subject of the most comprehensive research among them, but many recent studies have revealed that SIRT2 can play an important role in life events such as inflammation, apoptosis and cellular senescence through the control of gene transcription and that abnormalities in these life events may be closely linked to tumorigenesis and development (36, 37). According to previous studies, SIRT2 is involved in the development and progression of many types of cancer, and it may have a greater impact on individuals than SIRT1 (38). Renal cancer cells express high levels of SIRT2, which is associated with a poorer prognosis for patients (39). McGlynn et al. found that high protein levels of nuclear SIRT2 were differentially associated with the recurrence of different grades of breast cancer (40). Therefore, the controversy makes SIRT2 a very interesting candidate for further tumor research.

In this work, TCGA data online and independent datasets were used to determine SIRT2 expression levels and construct systematic prognostic landscapes for different types of lung cancer. Various types of cancer have also been examined for variation in SIRT2 expression levels between cancer and normal tissues. By comparing normal tissues using the TIMER2.0 database, we found high levels of SIRT2 expression in tumor tissues of CHOL, ESCA, KICH, KIRC and LIHC, while the converse is true in the case of KIRP, LUAD, LUSC, STAD and UCEC. However, compared with paired adjacent non-tumor tissues, the redetermination of the TCGA data revealed SIRT2 was highly expressed in CHOL, HNSC, KICH, KIRC, and LIHC, but significantly lower in BRCA, KIRC, LUAD, UCEC, and slightly lower in LUSC tissue.

Moreover, LGG is the only type of cancer in which the SIRT2 expression level is higher than the paired normal tissue, while SIRT2 is lowly expressed in OV, TGCT, and UCEC based on the GEPIA database. The expression levels of SIRT2 in multiple cancers may differ from database to database, which may depend on the underlying biological mechanisms and the data collection approaches, and also the comparison criterion might be a reason.

Nevertheless, in the remaining databases (GEPIA, TIMER2.0, and Oncolnc), we found an association between SIRT2 expression and the prognosis of HNSC, LUAD and OV. Among these three types of cancer, our results show that SIRT2 expression levels are positively correlated with prognosis in the HNSC and LUAD, and negatively in the OV cohort. Furthermore, by analysing the patient cohorts in the PrognoScan database and Kaplan-Meier Plotter, we found that high SIRT2 expression level was associated with better prognosis (OS or DFS) in brain, colorectal, and lung cancer, while the prognosis in the bladder, breast, and ovarian cancers was the opposite. Two PrognoScan datasets showed that higher SIRT2 expression levels were predictive of a better outcome in LUAD. Moreover, high levels of SIRT2 expression were associated with a better early prognosis in LUAD, with a better HR [0.41 (0.23-0.74)] when SIRT2 was highly expressed in LUAD but not in LUSC. This collectively suggests that SIRT2 is a potential prognostic biomarker in LUAD.

Another important finding was the correlation between the level of SIRT2 expression and the level of cancer-associated immune infiltration, particularly in LUAD. Here, our results show the infiltration level of T cells (CD4+ T cells and CD8+ T cells), neutrophils, macrophages, and DCs in LUAD is strongly positively correlated with SIRT2 expression. Additionally, the correlation pattern of infiltrating level and SIRT2 expression differs between LUAD and LUSC. According to the association between SIRT2 expression and tumor immune infiltration, we speculate that SIRT2 may be involved in regulating tumor immunology. One possible reason for this particular phenomenon is that the gene expression product of SIRT2 is involved in the coordination of the function of several immune marker gene sets. On this basis we propose the thesis that aberrant expression of SIRT2 is an important contributor to the development of a variety of malignancies and has the prognostic potential for specific types of cancer.

Firstly, M1 macrophages show a negative correlation with SIRT2 expression in LUSC rather than in LUAD, respectively (**Table 2**). Since macrophages have important tumor immunological functions, M1 macrophages produce type 1 cytokines that prevent tumor development, and conversely, M2 macrophages induce the production of type 2 cytokines which promote tumor growth. These results suggest that SIRT2 may have a potential regulatory role in tumorigenesis and development through its involvement in macrophage polarization. Through alterations in the microenvironment, SIRT2 may repolarize activated macrophages to opposite functional phenotypes, resulting in opposite effects on tumorigenesis.

**Table 2:** Molecular docking study of the Resveratrol and analogs with SIRTs (PDB: 5D7N).

X	Y	Z	Compound	Structural formula	Binding energy (kcal/mol)	Inhibition constant (Ki)
OH	OH	OH	Resveratrol	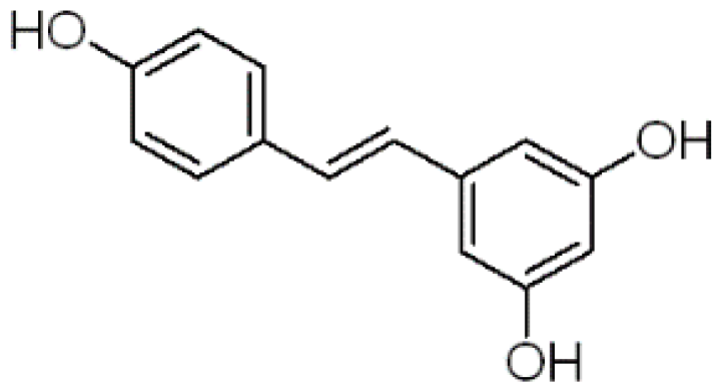	-7.62	2.61 uM
OH	OH	OH	Dihydroresveratrol	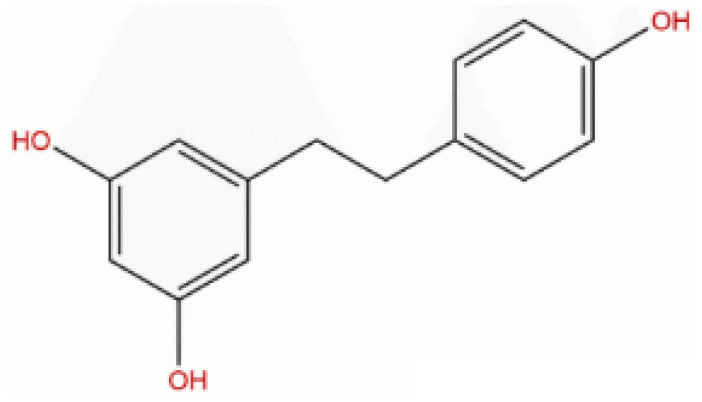	-7.55	2.92 uM
F	OH	OH	Resveratrol analog	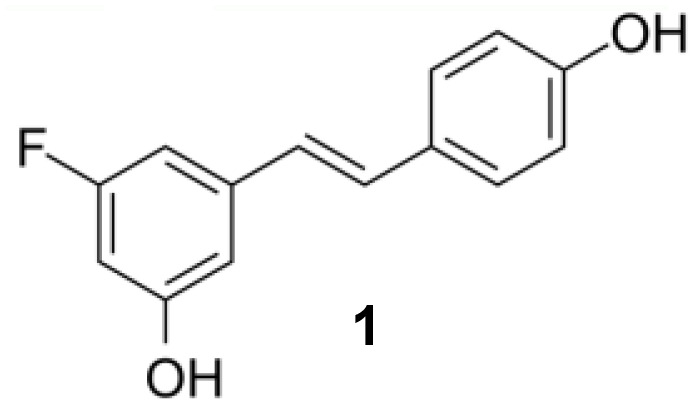	-7.28	4.59 uM
F	OH	OAc	Resveratrol analog	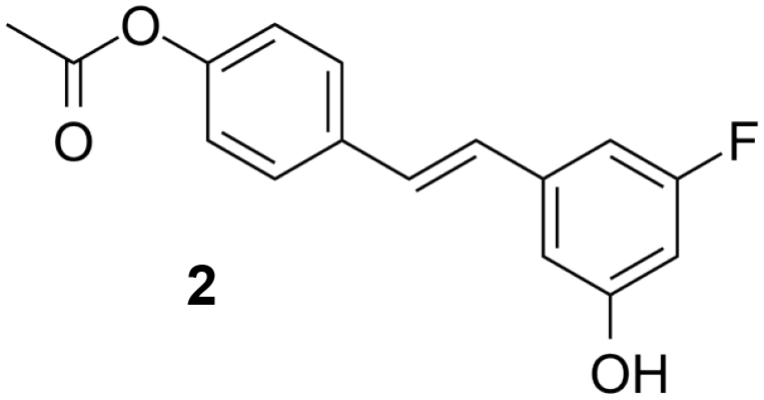	-7.93	1.54 uM
OAc	OAc	OAc	Resveratrol analog	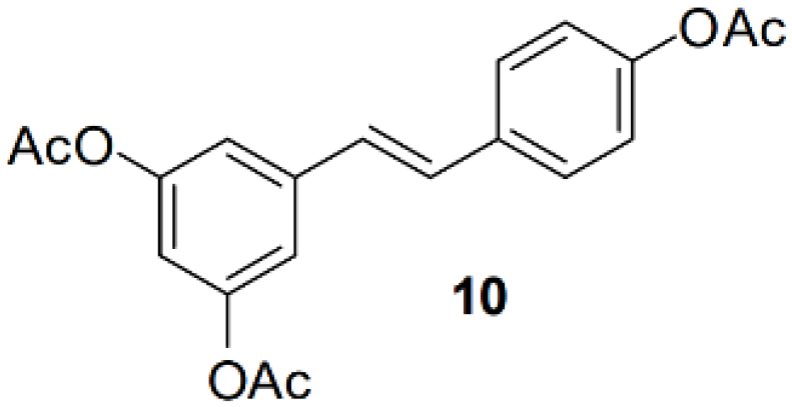	-9.34	142.79 nM
OH	OH	OAc	Resveratrol analog	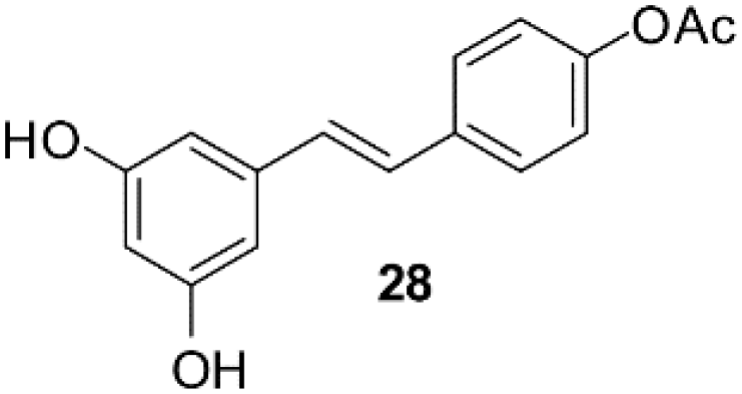	-7.91	1.60 uM
OAc	OAc	OH	Resveratrol analog	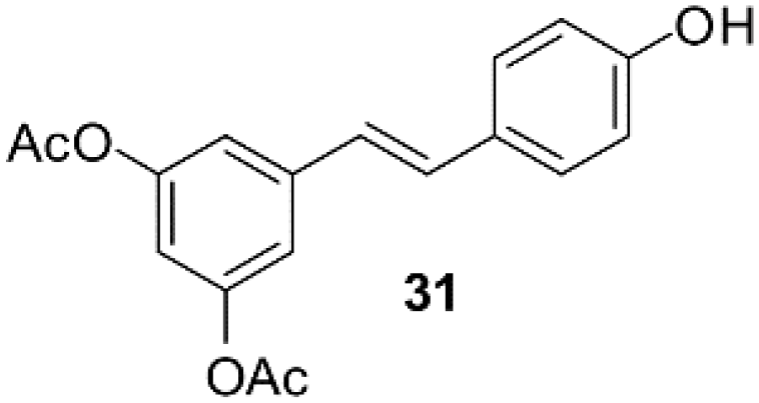	-8.81	348.00 nM
OH	OAc	OAc	Resveratrol analog	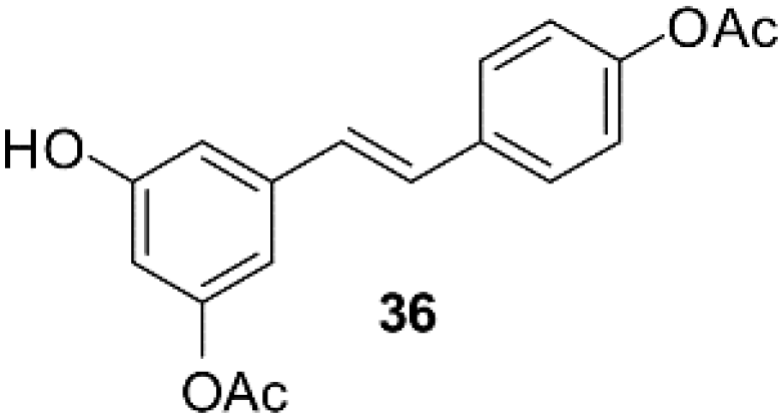	-8.66	451.80 nM
OH	OAc	OH	Resveratrol analog	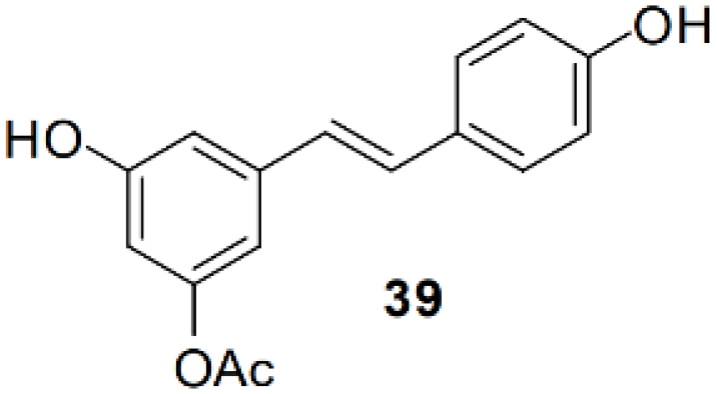	-8.32	795.19 nM

Secondly, It appears that SIRT2 is capable of activating different types of T cells (CD8+ T cells, naive T cells, effector T cells) as well as natural killer cells, inactivating Tregs. CD8A, an important glycoprotein on the surface of T cells involved in intercellular interactions in the immune response, is highly correlated with SIRT2 expression in LUAD which are types of cancers with better prognosis. Furthermore, CD8A did not show a clear pattern of correlation in LUSC. This pattern of correlation with SIRT2 gene expression is also seen in general T cell markers (eg, CD3D, CD3E, CD2) and in some other immune cell populations (eg, most naive T cell markers, effector memory T cells, effector T cells, and natural killer cells), such as LEF1 which has been shown to predict variations in response to treatment in AML.

Thirdly, in tumor and normal tissues of LUAD and LUSC, the levels of SIRT2 expression showed different patterns of correlation with the regulation of various immune cells (eg, Th1, central memory T cells, and resident memory T cells). For example, Th1 has both pro- and anti-cancer effects in different human cancers, it is highly correlated with SIRT2 expression levels in LUAD while in LUSC there are no significant correlations.

Thus, these explanations specifically explain why high SIRT2 expression represents a better prognosis in LUAD but is not significantly correlated to LUSC.

The AcO group of triacetylresveratrol binds to SIRT2 in a cavity through hydrogen bonding with Ile169 and Leu103 of SIRT2, van der Waals contact with Thr101, and forms a salt bridge to Arg97 (**Figure 7C**), which is not found in the interactions between Resveratrol and SIRT2, although the hydroxyphenyl group of Resveratrol forms hydrogen bonds with residues Phe96, ASP170, and Ala135 and van der Waals interactions with Asp96 and Asn168 (**Figure 7A**). Interestingly, the introduction of the F group in Resveratrol analog 2 also apparently attracts positively charged residues (Arg97 and His187) to build salt bridges (**Figure 7B**), which strongly binds the agonist in the pocket, but also hinders the interaction of distal functional group (i.e. AcO). Additionally, a p-π interaction between AcO group of Resveratrol analog 10 and Phe190 is observed, whereas, in SIRT2-resveratrol and SIRT2-resveratrol analog 2, Phe190 forms π-π and H-π interactions with phenyl ring of agonists, respectively. Notably, the agonist, Resveratrol analog 10, appears to be surrounded by hydrophobic residues, i.e. Ile93, Pro94, Phe96, and Leu138, compared with the hydrophobic cluster of Ile93 and Ile169 for SIRT2-resveratrol. Therefore, although the excessive salt bridge interaction for the F group will block the binding of the distal functional group, the substitution of the hydroxyl group of Resveratrol with the AcO group and the F group will form salt bridges with Arg97 and/or His187 and improve the hydrophobic interaction to increase the binding affinity of the agonist, more potentially preventing its activation-related motion thus stabilizing the receptor in an inactive conformation, in agreement with a decrease in antagonist affinity when the carbamoyl group was replaced by an alkoxycarbonyl, acyl or alkyl group21.

## Conclusions

In this study, we proposed a possible hypothesis about why SIRT2 expression levels correlate with immune infiltration levels as well as prognosis in LUAD and LUSC, that is the recruiting and activating TILs *via* SIRT2. These results provide insight into the possible role of SIRT2 in tumor immunology and its application as a prognostic biomarker and as a novel therapeutic target for LUAD. So we believe that SIRT2 agonists could be immunomodulators of LUAD to immunotherapy combination therapies, and triacetylresveratrol would be a novel, potent activity agonist for SIRT2 than resveratrol.

## Data availability statement

The original contributions presented in the study are included in the article/**Supplementary Material**. Further inquiries can be directed to the corresponding authors.

## Author contributions

JH contributed to the idea, conception and study design. JH and NQ collected and analyzed the datasets. ZS and JL conducted the analysis of the data, ZL and XZ verified the result of the study. JH, MM, and NQ wrote the manuscript and generated the figures. SD and HW revised and proofread the article. All authors contributed to the article and approved the submitted version.
