# Supplementary material for: Resveratrol analog, triacetylresveratrol, a potential immunomodulator of lung adenocarcinoma immunotherapy combination therapies
Source: Front Oncol. 2023 Feb 9;12:1007653. doi: 10.3389/fonc.2022.1007653 (PMC9947150; doi:10.3389/fonc.2022.1007653)
Supplement: Supplementary file 4 [file Presentation_1.pptx]

## Slide 1
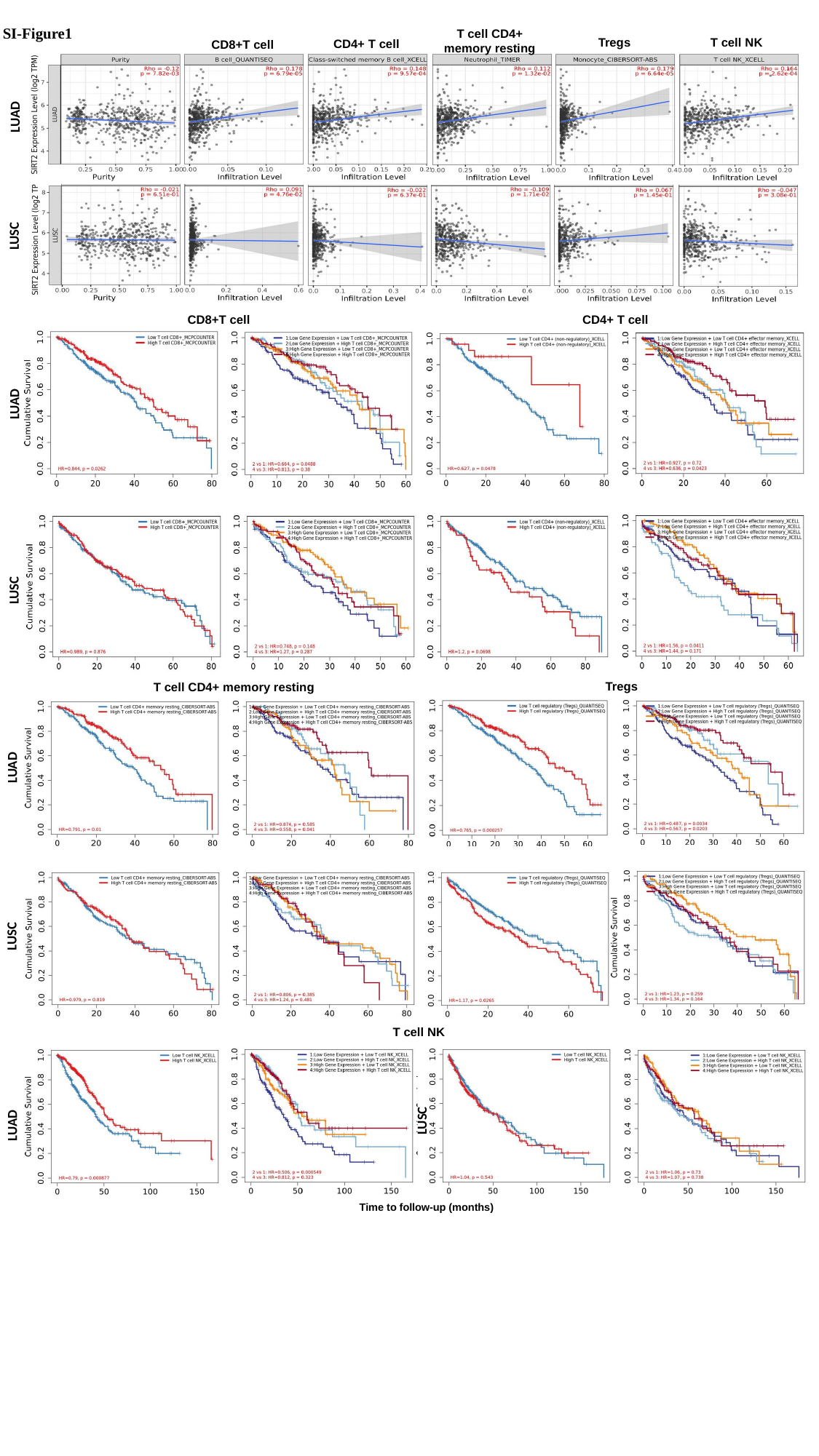

SI-Figure1
T cell CD4+ memory resting
T cell NK
Tregs
CD4+ T cell
CD8+T cell
LUAD
LUSC
CD8+T cell
CD4+ T cell
LUAD
LUSC
Tregs
T cell CD4+ memory resting
LUAD
LUSC
T cell NK
LUAD
LUSC
Time to follow-up (months)
