# Supplementary material for: Resveratrol analog, triacetylresveratrol, a potential immunomodulator of lung adenocarcinoma immunotherapy combination therapies
Source: Front Oncol. 2023 Feb 9;12:1007653. doi: 10.3389/fonc.2022.1007653 (PMC9947150; doi:10.3389/fonc.2022.1007653)
Supplement: Supplementary file 5 [file Presentation_2.pptx]

## Slide 1
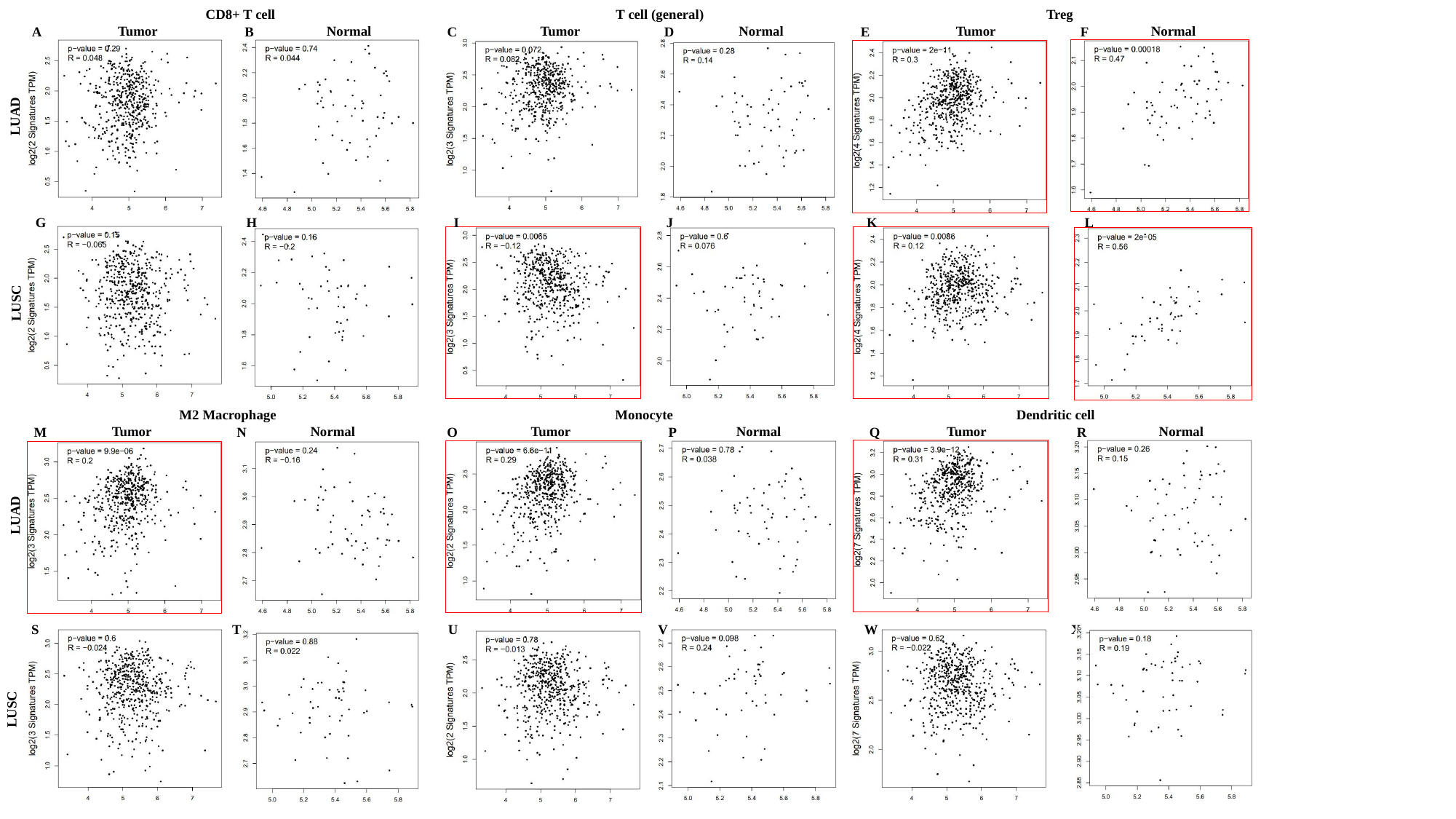

CD8+ T cell
T cell (general)
Treg
Tumor Normal Tumor Normal Tumor Normal
A B C D E F
LUAD
G H I J K L
LUSC
M2 Macrophage
Monocyte
Dendritic cell
Tumor Normal Tumor Normal Tumor Normal
M N O P Q R
LUAD
S T U V W X
LUSC
log2(SIRT2 TPM)
